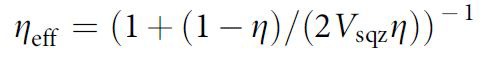# Erratum: Quantum enhanced feedback cooling of a mechanical oscillator using nonclassical light

**DOI:** 10.1038/ncomms14489

**Published:** 2017-02-03

**Authors:** Clemens Schäfermeier, Hugo Kerdoncuff, Ulrich B. Hoff, Hao Fu, Alexander Huck, Jan Bilek, Glen I. Harris, Warwick P. Bowen, Tobias Gehring, Ulrik L. Andersen

Nature Communications 7: Article number: 13628; DOI: 10.1038/ncomms13628 (2014); Published 11 29 2016; Updated 12 03 2017

This Article contains a typographical error in the second line of the right column on page 4, in which the exponent ‘−1' incorrectly appears before the final closing parenthesis. The correct equation is