# Supplementary material for: Quantum enhanced feedback cooling of a mechanical oscillator using nonclassical light
Source: Nat Commun. 2016 Nov 29;7:13628. doi: 10.1038/ncomms13628 (PMC5141296; doi:10.1038/ncomms13628)
Supplement: Supplementary Information — Supplementary Figures 1-9, Supplementary Notes 1-2, Supplementary Methods and Supplementary References. [file ncomms13628-s1.pdf]

# I. SUPPLEMENTARY FIGURES

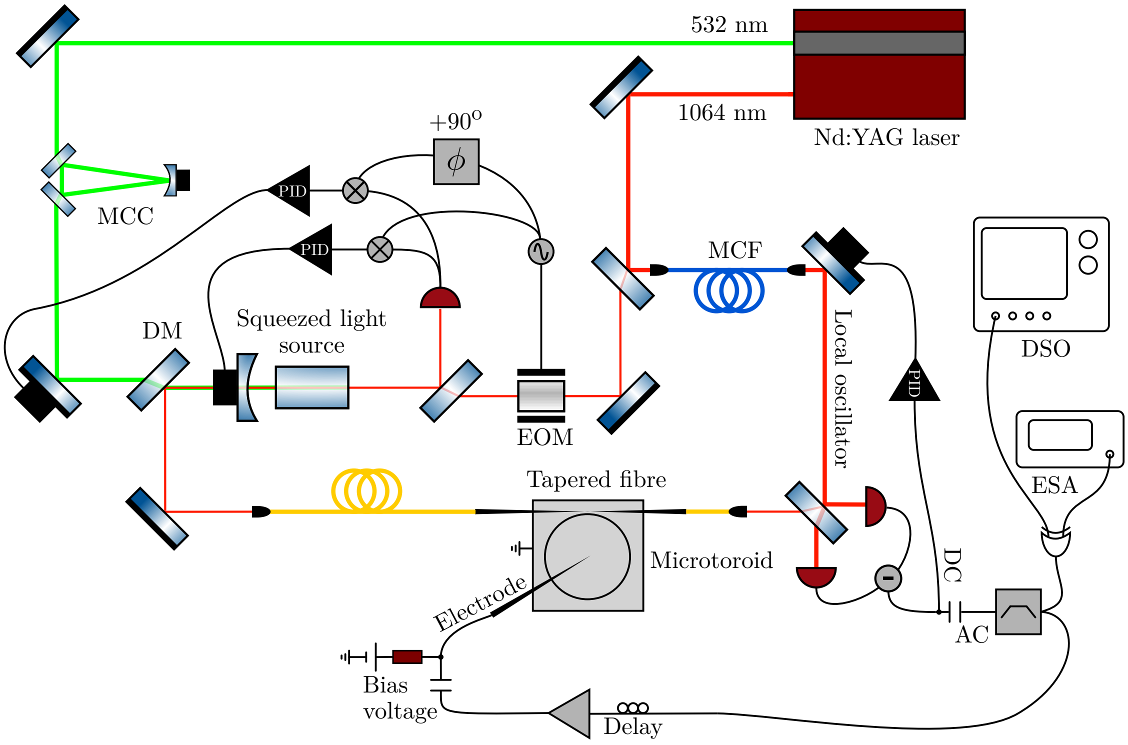


Supplementary Figure 1. Schematic representation of the experimental setup. MCC: mode cleaning cavity, EOM: electro-optic modulator, MCF: mode cleaning fibre, DM: dichroic mirror, ESA: electronic spectrum analyser, DSO: digital sampling oscilloscope, PID: servomechanism, AC / DC: alternating / direct current, Nd:YAG: neodymium-doped yttrium aluminium garnet.

(a)

(b)

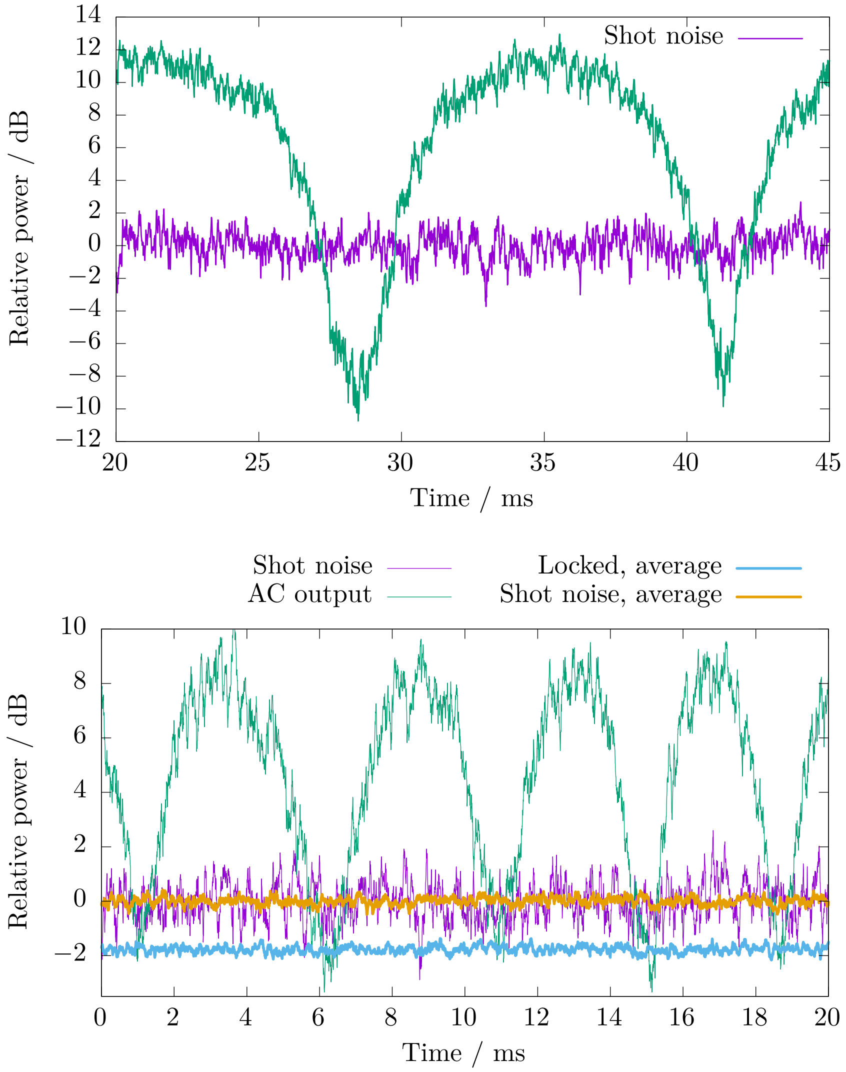


Supplementary Figure 2. Homodyne tomography of the squeezed state. Recorded (a) immediately after the source and (b) after transmission through the tapered fibre. A local oscillator power of 12 mW and 4.25 mW was used, respectively. In either case the electronic noise (more than 13 dB below shot noise level) has been subtracted. All data was acquired with a resolution bandwidth of 300 kHz and a video bandwidth of 3 kHz. Traces were recorded five times when averaging was applied.

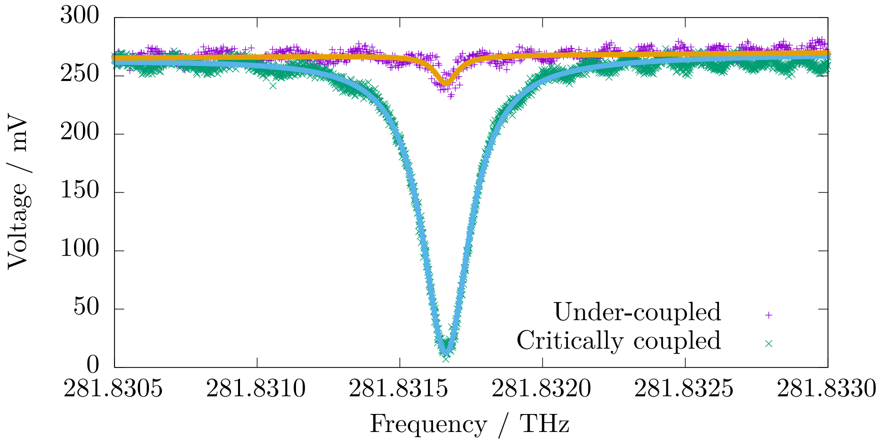


Supplementary Figure 3. Transmission spectra of the optical resonance of the microtoroidal resonator. Recorded for the case of near-critical coupling and under coupling. The laser-frequency dependent modulation observed in the off-resonant parts of the spectra are attributed to parasitic cavity effects in the setup. The contribution to this effect from the tapered fibre was mitigated by using FC/APC fibre connectors.

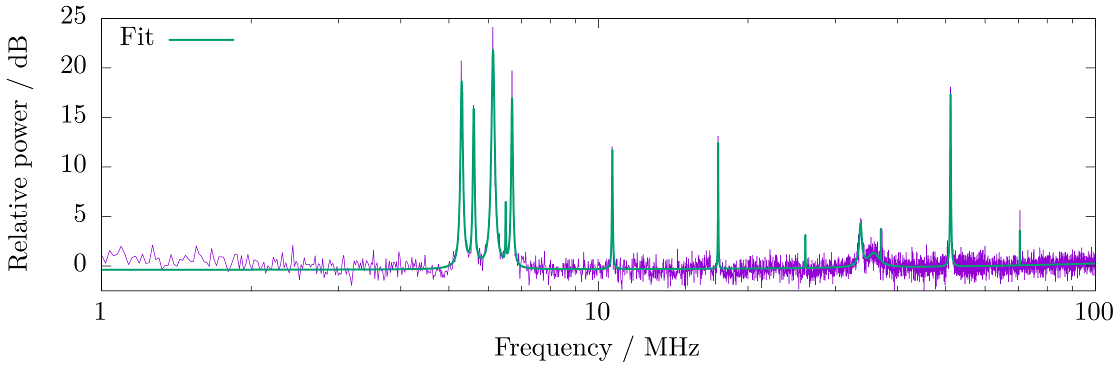


Supplementary Figure 4. Power spectrum of the transduced mechanical modes. The fundamental flexural mode used for cooling appears at a frequency of 6.13 MHz. Dark noise was subtracted from the recording. The shot-noise power spectral density was taken as the reference level.

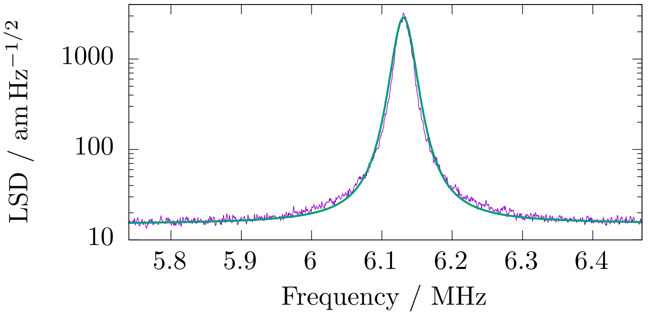


Supplementary Figure 5. Linear spectral density (LSD) of the mechanical fundamental flexural mode. This mode was chosen for feedback cooling and was calibrated to the actual mechanical displacement amplitude.

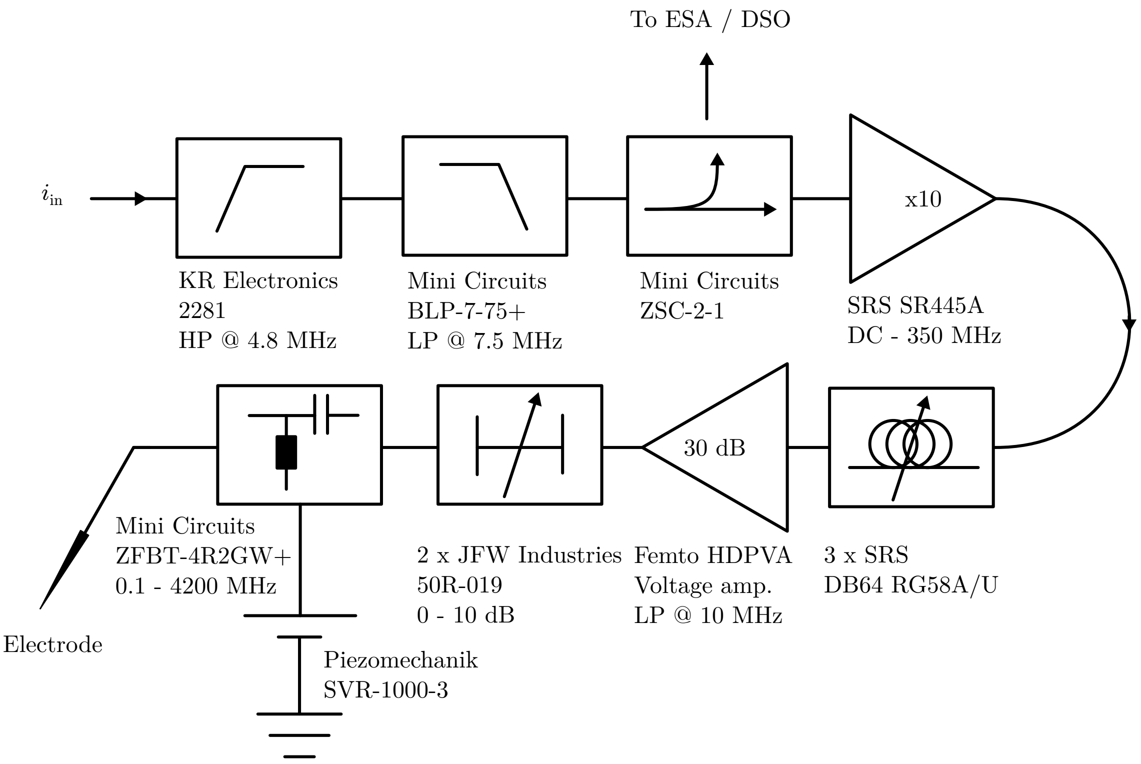


Supplementary Figure 6. Schematic representation of the implemented electronic feedback circuit. $i_{\text{in}}$: AC-coupled homodyne photocurrent, ESA: electronic spectrum analyser, DSO: digital sampling oscilloscope. From input to output: high-pass filter, low-pass filter, splitter, amplifier, variable delay line, amplifier, variable attenuator, bias-tee.

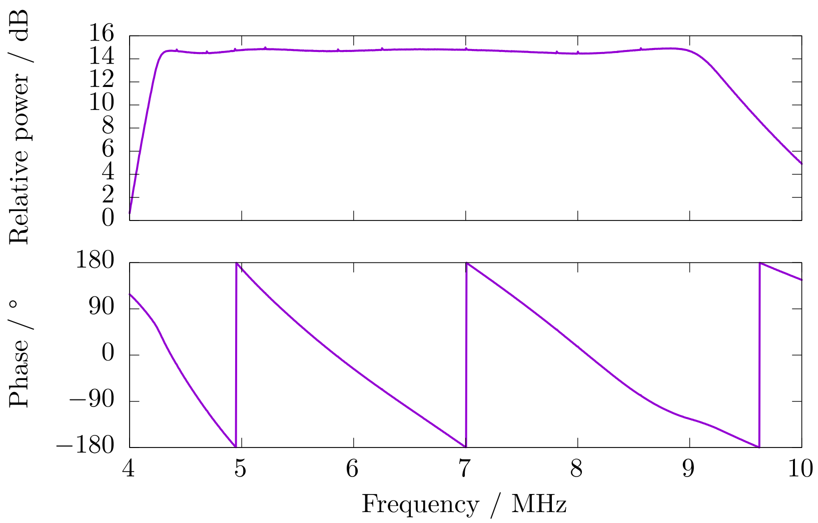


Supplementary Figure 7. The forward voltage gain ($S_{21}$) transfer function of the total feedback circuit.

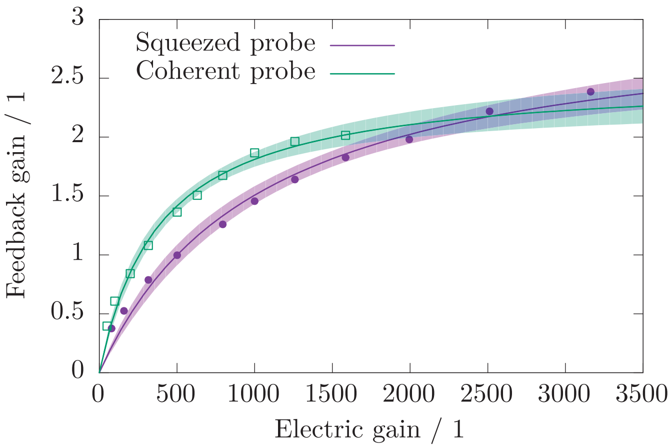


Supplementary Figure 8. Feedback gain ($G_{\mathrm{Fb}}$) as a function of the set electric gain for both probe states. Higher gain settings were chosen for the squeezed probe, as more gain is required to reach the optimal temperature at a higher initial SNR. A shading around the solid lines represents the 95 % confidence level of the prediction band. The calibration is discussed in Supplementary Method III E.

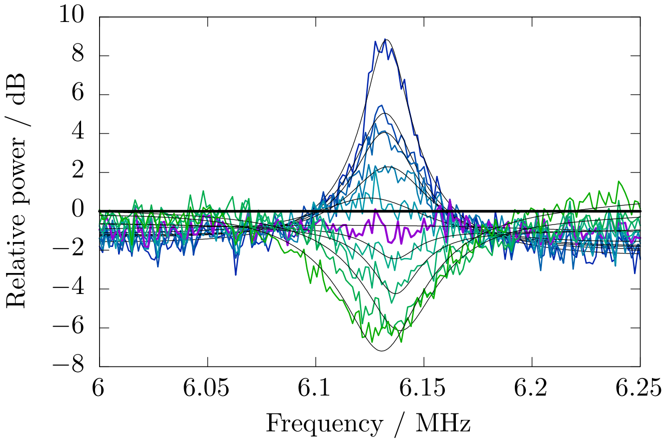


Supplementary Figure 9. Spectral densities recorded with various gain settings. Applying gain settings beyond the point which yields the lowest temperature leads to squashing. This is a regime where the correlations between electronic noise and the mechanical oscillator dominate the system. The spectra were normalised to the optical shot noise level.

## II. SUPPLEMENTARY NOTES

### A. Supplementary Note 1: Feedback cooling of a microtoroid resonator

Feedback cooling, also known as cold damping, relies on the sensitive measurement of mechanical motion to apply an active negative feedback, which increases the damping of the mechanical oscillator [1]. The optimal cooling achievable with a feedback cooling scheme is usually limited by the measurement noise and the feedback gain. Cavity optomechanical systems allow ultrahigh-sensitivity measurements of mechanical motion due to the high frequencies of optical fields and the cavity-enhanced optomechanical interaction. Cooling can then be achieved by feeding back the measured low noise signal to a control force actuating the mechanical oscillator. The radiation pressure force can be used for this purpose [2–4], but it remains inherently weak and the optical powers required to significantly actuate the mechanical motion may actually lead to heating, especially for cryogenically pre-cooled systems. Alternative actuation schemes involve piezo-driven cantilevers [5], coil actuators [6, 7], optical tweezers [8], and dielectric gradient forces [9].

We consider a mechanical oscillator, fundamentally subject to three external forces: a thermal Brownian force $\hat{F}_{\mathrm{th}}(\Omega)$ due to the ambient environment, a radiation pressure back-action force $\hat{F}_{\mathrm{ba}}(\Omega)$ associated with coupling of the oscillator to an optical probe field, and finally a feedback actuation force $\hat{F}_{\mathrm{Fb}}(\Omega)$. For the system considered in the manuscript the radiation pressure force is much weaker than the thermal Brownian force and remains hidden below the measurement noise. Thus, this contribution will henceforth be neglected. The effect of back-action has only recently been observed in state of the art experiments on macroscopic oscillators [10–12].

The applied feedback force acts to impose a viscous damping of a particular frequency component of the mechanical motion. This is achieved by continuously measuring the oscillators displacement, $\hat{x}(\Omega)$, and applying a force proportional to the measurement delayed by a quarter of the mechanical oscillation period. Assuming a harmonic mechanical oscillation the applied feedback force is proportional to the instantaneous velocity, realising an effective viscous damping.

The actual feedback force is given by

$$\hat{F}_{\mathrm{Fb}}(\Omega) = m_{\mathrm{eff}} K \left(\hat{x}(\Omega) + \hat{x}_{\mathrm{n}}(\Omega)\right), \tag{1}$$

where the first term is proportional to the continuous measurement outcome while the second is a stochastic term due to imprecision noise $\hat{x}_{\mathrm{n}}(\Omega)$ of the displacement measurement, including all noise sources in the feedback loop, e.g. optical shot noise, detection noise, and electronic noise. The pre-factor is composed of an effective mass of the oscillation mode, $m_{\mathrm{eff}}$, and a (generally complex

valued) transfer function, $K$, characterising gain and phase lag of the feedback loop.

The frequency-domain response of the oscillator to external forces is characterised by the susceptibility $\chi_{\mathrm{m}}(\Omega) = \left[(\Omega_{\mathrm{m}}^2 - \Omega^2) - i\Omega\Gamma_{\mathrm{m}}\right]^{-1}$, where $\Omega_{\mathrm{m}}$ and $\Gamma_{\mathrm{m}}$ is the resonance frequency and damping rate, respectively. The resulting displacement is given by

$$\begin{aligned}\hat{x}(\Omega) &= m_{\mathrm{eff}}^{-1}\chi_{\mathrm{m}}(\Omega)\left(\hat{F}_{\mathrm{th}}(\Omega) + \hat{F}_{\mathrm{Fb}}(\Omega)\right) \\ &= m_{\mathrm{eff}}^{-1}\chi_{\mathrm{m}}(\Omega)\left(\hat{F}_{\mathrm{th}}(\Omega) + m_{\mathrm{eff}}K\left(\hat{x}(\Omega) + \hat{x}_{\mathrm{n}}(\Omega)\right)\right) \\ &= m_{\mathrm{eff}}^{-1}\chi_{\mathrm{eff}}(\Omega)\left(\hat{F}_{\mathrm{th}}(\Omega) + m_{\mathrm{eff}}K\hat{x}_{\mathrm{n}}(\Omega)\right), \end{aligned} \tag{2}$$

where we have introduced an effective susceptibility:

$$\chi_{\mathrm{eff}}(\Omega) = \frac{\chi_{\mathrm{m}}(\Omega)}{1 - K\chi_{\mathrm{m}}(\Omega)} \tag{3}$$

$$= \left[(\Omega^2 - \Re(K) - \Omega_{\mathrm{m}}^2) - i\Omega\Gamma_{\mathrm{m}}(1 + \Im(K)/\Omega\Gamma_{\mathrm{m}})\right]^{-1}. \tag{4}$$

The real part of the feedback gain contributes to the spring constant of the mechanical oscillator, resulting in a feedback modified resonance frequency, $\Omega_{\mathrm{Fb}}^2 = \Omega_{\mathrm{m}}^2 - \Re(K)$, while the imaginary part contributes to the dissipation of the mechanical oscillator, leading to a feedback induced damping rate, $\Gamma_{\mathrm{Fb}} = \Gamma_{\mathrm{m}}(1 + G_{\mathrm{Fb}})$, with $G_{\mathrm{Fb}} = \Im(K)/\Omega\Gamma_{\mathrm{m}}$. In more physical terms, this means that the stiffening or softening of the mechanical oscillator, as well as its dissipative coupling to the environment, can be controlled by designing an appropriate feedback transfer function. Generally, the feedback transfer function can be expressed as

$$K = G_{\mathrm{Fb}}\Omega\Gamma_{\mathrm{m}}\exp(i\Omega_{\mathrm{m}}\tau_{\mathrm{Fb}}), \tag{5}$$

where $\tau_{\mathrm{Fb}}$ is the delay of the feedback loop. Henceforth, we will assume $\Omega_{\mathrm{m}}\tau_{\mathrm{Fb}} = \pi/2 \mod 2\pi$, corresponding to an ideal dissipative feedback force.

### 1. *Displacement spectrum of the feedback actuated resonator*

The power spectral density (PSD) for an operator $\hat{O}$ is generally defined as

$$S_O(\Omega) = \langle|\hat{O}(\Omega)|^2\rangle. \tag{6}$$

Using Supplementary Equation (2) and assuming that the noise terms are uncorrelated the mechanical displacement PSD is given by

$$S_x(\Omega) = \langle|\hat{x}(\Omega)|^2\rangle = \frac{|\chi_{\mathrm{eff}}(\Omega)|^2}{m_{\mathrm{eff}}^2}\left(S_{F_{\mathrm{th}}}(\Omega) + m_{\mathrm{eff}}^2|K|^2 S_{x_{\mathrm{n}}}(\Omega)\right). \tag{7}$$

Inserting Supplementary Equation (4) and (5) yields the following PSD for the mechanical displacement subject to feedback:

$$S_x(\Omega) = \left[\frac{1/m_{\mathrm{eff}}^2}{(\Omega_{\mathrm{m}}^2 - \Omega^2)^2 + ((1+G_{\mathrm{Fb}})\Omega_{\mathrm{m}}\Omega/Q_{\mathrm{m}})^2}\right] S_{F_{\mathrm{th}}}(\Omega) + \left[\frac{(\Omega G_{\mathrm{Fb}}\Omega_{\mathrm{m}}/Q_{\mathrm{m}})^2}{(\Omega_{\mathrm{m}}^2 - \Omega^2)^2 + ((1+G_{\mathrm{Fb}})\Omega_{\mathrm{m}}\Omega/Q_{\mathrm{m}})^2}\right] S_{x_{\mathrm{n}}}(\Omega), \tag{8}$$

where $Q_{\mathrm{m}}$ denotes the quality factor of the mechanical oscillator. Here, the first term represents driving of the mechanical oscillator by the environment Brownian thermal noise while the second term represents the mechanical position uncertainty introduced by imprecision noise in the feedback loop being fed back onto the oscillator.

Supplementary Equation (8) reflects the actual displacement of the feedback actuated oscillator, that is the displacement as it would be revealed by an ideal out-of-loop measurement. An in-loop measurement, on the other hand, includes additional measurement imprecision noise and is given by $\hat{x}(\Omega) + \hat{x}_{\mathrm{n}}(\Omega)$ with a PSD of

$$S_x^{\mathrm{meas}}(\Omega) = \left[\frac{1/m_{\mathrm{eff}}^2}{(\Omega_{\mathrm{m}}^2 - \Omega^2)^2 + ((1+G_{\mathrm{Fb}})\Omega_{\mathrm{m}}\Omega/Q_{\mathrm{m}})^2}\right] S_{F_{\mathrm{th}}}(\Omega) + \left[\frac{(\Omega_{\mathrm{m}}^2 - \Omega^2)^2 + (\Omega\Omega_{\mathrm{m}}/Q_{\mathrm{m}})^2}{(\Omega_{\mathrm{m}}^2 - \Omega^2)^2 + ((1+G_{\mathrm{Fb}})\Omega_{\mathrm{m}}\Omega/Q_{\mathrm{m}})^2}\right] S_{x_{\mathrm{n}}}(\Omega). \tag{9}$$

It is important to distinguish the above out-of-loop (8) and in-loop (9) measurement spectra as they suggest vastly different behaviour of the mechanical oscillator as function of feedback gain. In case of an out-of-loop measurement, two independent transduction measurements, e.g. detuned laser sources, are used for feedback actuation and displacement spectrum characterisation. Consequently, the actual displacement of the cooled oscillator and hence its temperature can be inferred. For an in-loop measurement, however, one and the same transduction measurement is used both for characterisation and to generate the feedback force. As the feedback gain is increased, the oscillator motion is progressively correlated with the inevitable imprecision noise of the measurement, resulting in an artificial noise suppression – *squashing* – in the measured displacement spectrum. For sufficiently large gain values the measured spectrum even suggests a mechanical noise below the measurement noise level. As discussed in the following section, the effective mode temperature is related to the integral of the displacement spectrum. In the presence of noise squashing this leads to inference of negative temperatures which is obviously unphysical. This means that care should be taken when inferring the feedback cooled oscillator temperature from in-loop measurements as considered in the main manuscript.

### 2. *Effective temperature of the feedback actuated resonator*

According to the fluctuation-dissipation theorem we may define an effective temperature from the PSD of the mechanical position fluctuations. The effective temperature $T_{\mathrm{Fb}}$ of a mechanical oscillator subject to a feedback actuation force can be calibrated relative to the effective temperature $T$ of the same oscillator in thermal equilibrium with its environment. Assuming that the PSD of the position fluctuations under feedback actuation retains a Lorentzian profile with effective resonance frequency $\Omega_{\mathrm{Fb}}$ and damping rate $\Gamma_{\mathrm{Fb}}$, the effective temperature of the oscillator is given by

$$\frac{T_{\mathrm{Fb}}}{T} = \frac{\int_{-\infty}^{\infty}\langle|\hat{x}(\Omega)|^2\rangle \mathrm{d}\Omega}{\int_{-\infty}^{\infty}\langle|\hat{x}(\Omega)|^2\rangle_{G_{\mathrm{Fb}}=0}\mathrm{d}\Omega} = \frac{\Gamma_{\mathrm{Fb}}\langle|\hat{x}(\Omega_{\mathrm{Fb}})|^2\rangle}{\Gamma_{\mathrm{m}}\langle|\hat{x}(\Omega_{\mathrm{m}})|^2\rangle_{G_{\mathrm{Fb}}=0}}. \tag{10}$$

Substituting Supplementary Equation (2) into Supplementary Equation (10) the feedback induced temperature can be expressed in terms of experimentally accessible quantities as

$$\frac{T_{\mathrm{Fb}}}{T} = \left(1 + \frac{G_{\mathrm{Fb}}^2}{\mathrm{SNR}}\right)\frac{1}{1+G_{\mathrm{Fb}}}, \tag{11}$$

where we have introduced the signal-to-noise ratio (SNR) as the level of the on-resonance mechanical position noise relative to the off-resonance measurement noise level:

$$\mathrm{SNR} = \frac{\langle|\delta\hat{x}(\Omega_{\mathrm{m}})|^2\rangle_{G_{\mathrm{Fb}}=0}}{\langle|\delta\hat{x}_{\mathrm{noise}}(\Omega_{\mathrm{m}})|^2\rangle}, \tag{12}$$

The SNR can be determined directly from the PSD of the measured cavity output field fluctuations. For fixed detection conditions, the minimum temperature achievable by cold damping is limited to

$$T_{\mathrm{min}} = 2T\frac{\sqrt{1+\mathrm{SNR}}-1}{\mathrm{SNR}}, \tag{13}$$

for an optimised feedback gain of $G_{\mathrm{Fb}} = \sqrt{1+\mathrm{SNR}}-1$. At larger gain, $G_{\mathrm{Fb}} > \sqrt{1+\mathrm{SNR}}-1$, the driving of the mechanical oscillator by the measurement noise injected into the feedback loop overcomes the damping of the oscillations, and consequently the temperature rises with increased feedback gain. Therefore, it is essential to maximize the SNR in order to reach the largest possible cooling efficiency.

## B. Supplementary Note 2: Improving the detection efficiency by amplitude squeezing

In the strong measurement limit, where the rate of measurement dominates the mechanical decay rate, when implementing perfect feedback, the final position variance of the mechanical

oscillator is

$$V(\hat{q}) = \frac{1}{2\eta_{\text{eff}}} \tag{14}$$

in dimensionless units where the quantum zero-point motion variance equals one half, where $\eta_{\text{eff}}$ is the effective quantum efficiency of the measurement.

In general, the continuous measurement results in a photocurrent

$$i_{\text{in}}(t)dt = \sqrt{2\mu\eta_{\text{eff}}}\langle\hat{q}\rangle(t)\text{d}t + \text{d}W(t), \tag{15}$$

where $\mu$ is defined as the measurement rate and is determined not by the SNR of the measurement (as one might expect), but rather by the back-action the measurement introduces to the oscillator. Essentially, this is telling the total amount of information which is leaking out into the environment somehow due to the measurement. How much of this information you successfully collect is determined by $\eta_{\text{eff}}$. $\text{d}W(t)$ is a unit white-noise Wiener increment which accounts for the noise in the measurement.

It is easy to find that the SNR of the measurement is

$$\text{SNR} \equiv \frac{\langle i_{\text{in}}\rangle^2}{\langle i_{\text{in}}^2\rangle - \langle i_{\text{in}}\rangle^2} \propto 2\mu\eta_{\text{eff}}\langle\hat{q}\rangle^2(t). \tag{16}$$

For a coherent probe field $\eta_{\text{eff}} = \eta_{\text{d}}$, where $\eta_{\text{d}}$ is the detection efficiency (i.e. the probability that a photon within the optomechanical cavity is registered on the homodyne detector), and $\mu = C\Gamma_{\text{m}}$ with $C = 4g/\kappa\Gamma_{\text{m}}$ the optomechanical cooperativity. We therefore find that for a given expectation of the mechanical position the ratio of squeezed to coherent signal-to-noise ratios is

$$\frac{\text{SNR}_{\text{sqz}}}{\text{SNR}_{\text{coh}}} = \frac{\mu\eta_{\text{eff}}}{C\Gamma_{\text{m}}\eta_{\text{d}}}, \tag{17}$$

where we have retained the symbols $\mu$ and $\eta_{\text{eff}}$ to refer to the squeezed scenario.

As outlined by Bowen and Milburn [13], the measurement rate can be defined in general as $\mu = \Gamma_{\text{m}}\bar{n}_{\text{ba}}$, where $\bar{n}_{\text{ba}}$ is the mean increase in mechanical occupancy due to back-action from the measurement. In the bad cavity limit, $\bar{n}_{\text{ba}} = 2CS_{XX}$, where $S_{XX}$ is the amplitude quadrature's power spectral density of the incident field, so that

$$\mu = 2\Gamma CS_{XX}. \tag{18}$$

An initially pure amplitude squeezed field, with squeezed variance $V_{\text{sqz}}$, is injected into the optomechanical system. However, prior to the cavity it experiences loss, with the efficiency of impinging on the cavity given by $\eta_{\text{in}}$. The cavity itself is assumed to be perfectly one-sided with

no losses. Including losses here would be relatively easy, but we consider the essential physics sufficiently well described without this additional mathematical complexity. After the cavity, the light experiences further loss, with a detection efficiency of $\eta_{\mathrm{d}}$ and a total efficiency from input to output of $\eta = \eta_{\mathrm{in}}\eta_{\mathrm{d}}$.

The power spectral density of the incident field will be

$$S_{XX} = \eta_{\mathrm{in}} V_{\mathrm{sqz}} + (1 - \eta_{\mathrm{in}})/2. \tag{19}$$

We therefore find that

$$\mu = \Gamma_{\mathrm{m}} C \left(2\eta_{\mathrm{in}} V_{\mathrm{sqz}} + 1 - \eta_{\mathrm{in}}\right). \tag{20}$$

Similarly, when measuring the phase quadrature of the optical field ($V(\hat{Y})$) which contains maximal information about the mechanical position but is anti-squeezed, the ratio of squeezed to coherent signal-to-noise ratios will be

$$\frac{\mathrm{SNR}_{\mathrm{sqz}}}{\mathrm{SNR}_{\mathrm{coh}}} = \frac{1}{\eta/(2V_{\mathrm{sqz}}) + 1 - \eta}, \tag{21}$$

so that from Supplementary Equation (17)

$$\eta_{\mathrm{eff}} = \frac{C\Gamma_{\mathrm{m}}\eta_{\mathrm{d}}}{\mu} \frac{1}{\eta/(2V_{\mathrm{sqz}}) + 1 - \eta} \tag{22}$$

$$= \frac{\eta_{\mathrm{d}}}{\left(2\eta_{\mathrm{in}} V_{\mathrm{sqz}} + 1 - \eta_{\mathrm{in}}\right)\left(\eta/(2V_{\mathrm{sqz}}) + 1 - \eta\right)}. \tag{23}$$

We see, therefore, that the use of squeezing modifies the effective efficiency of feedback cooling.

It is interesting to ask what $V_{\mathrm{sqz}}$ optimises the efficiency. After some work, this can be shown to occur at

$$V_{\mathrm{sqz}}^{\mathrm{opt}} = \frac{1}{2}\sqrt{\frac{\eta(1-\eta_{\mathrm{in}})}{\eta_{\mathrm{in}}(1-\eta)}}, \tag{24}$$

which gives

$$\eta_{\mathrm{eff}}^{\mathrm{opt}} = \frac{\eta_{\mathrm{d}}}{\left(\sqrt{\eta\eta_{\mathrm{in}}} + \sqrt{(1-\eta)(1-\eta_{\mathrm{in}})}\right)^2}. \tag{25}$$

This optimum is always higher than $\eta_{\mathrm{d}}$, and is equal to unity (i.e. perfect efficiency) for all $\eta_{\mathrm{d}}$ when $\eta_{\mathrm{in}} = 1$. As an example, we consider a coupling efficiency of 90 % and a detector efficiency of 40 % which leads to an optimal effective efficiency of $\eta_{\mathrm{eff}}^{\mathrm{opt}} = 60\,\%$ for a squeezed state with 6 dB amplitude squeezing. This results in cooling of the mechanical oscillator down to an occupancy of $n_{\mathrm{min}} = 0.15$ rather than $n_{\mathrm{min}} = 0.29$ achievable for coherent state cooling.

## III. SUPPLEMENTARY METHODS

In the following we describe the experimental setup used for demonstration of squeezed light enhanced feedback cooling. Also we provide details on the operation and performance of the involved equipment.

### A. Supplementary Method 1: Experimental setup

A schematic representation of the experimental setup is shown in Supplementary Figure 1 including optical setup, control electronics, feedback circuit, and data acquisition. The optical setup was sourced by a continuous-wave Nd:YAG laser (Innolight GmbH Diabolo) delivering a fundamental output at 1064 nm and a secondary beam at 532 nm produced by second harmonic generation from the fundamental. The second harmonic field was employed as pump for squeezed light generation and for increased mode matching efficiency it was spatially filtered by means of a triangular travelling wave mode cleaning cavity (MCC) prior to coupling into the squeezing cavity. The MCC was stabilised using a standard Pound–Drever–Hall (PDH) scheme employing the internal phase modulation of the laser for error signal generation. The fundamental beam was split into two, one serving as seed for generation of bright squeezed probe states while the second and most intense part was used as local oscillator for homodyne detection. For improved homodyne visibility the local oscillator was coupled into a polarisation maintaining mode cleaning fibre and recoupled into free space before being interfered with the probe field. In the seed beam, an electro-optic modulator was used to generate phase modulation sidebands for PDH stabilisation of the squeezing cavity resonance. A second feedback loop actuating the phase of the pump beam was used for locking the squeezed light source to amplification, i.e. phase squeezing.

To allow for evanescent optical coupling to the microtoroidal resonator, the produced bright squeezed state was coupled into an adiabatically tapered single mode fibre with a diameter at the taper waist of $< 1\,\mu\mathrm{m}$. For better mechanical stability, the fibre was terminated with an FC/APC plug on each side. It further prevents from standing waves and improves the surface quality of the cleaved fibre tip. The mode matching from free space to fibre was measured by back-seeding the tapered fibre and matching the output to the squeezing cavity. This yielded a mode matching efficiency of $\eta_{\mathrm{mm}}^{\mathrm{fibre}} = 98.5\,\%$. The transmittance through the fibre was measured to be $T = 59\,\%$. The tapered fibre was kept at a fixed position while the microtoroid was held on a three-axes closed loop piezo stage (Thorlabs NanoMax MAX311D/M) providing control of

the relative taper-toroid separation with nanometre resolution. The coupling efficiency to the microtoroid was controlled by changing (i) the relative position between the tapered fibre and the microtoroid, (ii) the polarisation of the incoupled beam, and (iii) the temperature of the toroid. The toroid temperature was actuated by means of a Peltier element underneath the chip and the temperature stabilised using a temperature controller.

The probe field was then coupled back to free space and superimposed with the local oscillator beam on a 50:50 beam splitter. The interference visibility was measured to be $\mathcal{V} = 99\,\%$, corresponding to a mode matching efficiency of $\eta_{\mathrm{vis}} = \mathcal{V}^2 = 98\,\%$, and to stabilise the homodyne detector for phase quadrature measurement the DC part of the photocurrent was used as error signal for a feedback loop controlling the phase of the local oscillator by means of piezo actuated mirror. Both output modes of the beam splitter were detected by photo diodes with $\eta_{\mathrm{qe}} > 99\,\%$ quantum efficiency.

### B. Supplementary Method 2: Squeezed light source

The squeezed-light source consisted of a linear Fabry–Pérot resonator enclosing a 10 mm periodically poled potassium titanyl phosphate (ppKTP) crystal with two flat end-facets. One end-facet was high-reflective coated for both wavelengths, the fundamental at 1064 nm and the pump at 532 nm, serving as end mirror for the resonator. The other end-facet was anti-reflective coated for both wavelengths. The coupling mirror was attached to a piezo-electric transducer and had a reflectivity of 90 % for 1064 nm and 20 % for 532 nm. The mirror had a radius of curvature of 20 mm and was placed 13 mm from the crystal. This yielded a full-width-half-maximum of about 80 MHz. To achieve phase matching, the non-linear crystal was attached to a Peltier element. A phase matching temperature was reached at 36.16 °C. The cavity was locked by a PDH phase modulation-demodulation technique at 37.22 MHz using a 525 µW seed beam launched into the cavity from the high-reflective mirror. The pump phase was locked using the same phase modulation as for the cavity lock, but with a demodulation phase of 90° with respect to the cavity lock error signal. To achieve phase squeezing the pump phase was locked to amplification of the seed beam.

### 1. *Squeezing efficiency*

To characterise the efficiency of the squeezed light source, homodyne tomography of the generated state was performed. Immediately after the source (Supplementary Figure 2(a)) a reduction of the shot noise level by $-8\,\mathrm{dB}$ was observed, and with coupling to the microtoroid this was reduced to $-2\,\mathrm{dB}$ at the output of the tapered fibre (Supplementary Figure 2(b)). In the absence of coupling to the microtoroid, the total transmission efficiency through the tapered fibre was measured to be 54 %, including 4 % Fresnel loss from each of the uncoated fibre facets. At the fibre to microtoroid coupling strength used for both cooling experiments and the above squeezing characterisations the measured total transmission through the tapered fibre was $\eta_{\mathrm{total}} = 48\,\%$. From theory, the corresponding loss reduced degree of squeezing is given by

$$V_{\mathrm{out}}^{(\mathrm{dB})} = 10 \log \left[ \eta_{\mathrm{total}} 10^{V_{\mathrm{in}}^{(\mathrm{dB})}/10} + (1 - \eta_{\mathrm{total}}) \right], \tag{26}$$

resulting in $V_{\mathrm{out}} = -2.25\,\mathrm{dB}$ for $V_{\mathrm{in}} = -8\,\mathrm{dB}$, consistent with the experimentally measured value.

## C. Supplementary Method 3: Characterisation of the microtoroidal resonator

### 1. *Optical mode*

To characterise the used optical mode of the microtoroid we employed a secondary external cavity diode laser (Newport Velocity TLB-6721), tunable from 1050 nm to 1067 nm. Based on a Littman–Metcalf configuration the laser frequency was controlled by means of piezo-electric tuning of the external cavity feedback mirror. Using an control-voltage input, the laser frequency was tuned by $10\,\mathrm{GHz}\,\mathrm{V}^{-1}$. The frequency-swept laser was coupled to the microtoroid optical resonance by means of the tapered fibre and the resulting transmission spectrum was recorded by direct photo detection of the transmitted laser power. Examples of transmission spectra for different coupling to the optical resonance at $\omega_{\mathrm{c}}/2\pi = 281.83\,\mathrm{THz}$ are shown in Supplementary Figure 3. At near-critical coupling a Lorentzian fit to the transmission spectrum yields a FWHM resonance linewidth of $\Gamma_{\mathrm{c}}^{\mathrm{cc}}/2\pi = 204\,\mathrm{MHz}$ while in the strongly under-coupled case, relevant to the reported cooling experiments, the resulting linewidth is $\Gamma_{\mathrm{c}}^{\mathrm{uc}}/2\pi = 94.4\,\mathrm{MHz}$. The corresponding optical quality factors are $Q^{\mathrm{cc}} = 1.38 \cdot 10^6$ and $Q^{\mathrm{uc}} = 2.99 \cdot 10^6$, respectively.

For the feedback-cooling experiments an Nd:YAG laser with noise characteristics superior to the Newport Velocity TLB-6721 was used. However, the laser had a limited frequency tuning range of $\pm 200\,\mathrm{MHz}$, insufficient to cover the $> 1\,\mathrm{THz}$ free spectral range of the microtoroid optical

mode spectrum. To compensate for this, the microtoroid was mounted on a Peltier element to enable thermal tuning of its optical resonance frequency into the range of the Nd:YAG laser. A controllable incandescent light source in the microscope over the toroid enabled a fast temperature tuning.

### *2. Mechanical mode spectrum*

The transduced mechanical motion, imprinted on the optical probe field as a phase modulation in the case of resonant probing, was recorded by homodyne detection of the probe field. A typical spectrum of the transduced mechanical motion for the particular microtoroidal resonator is plotted in Supplementary Figure 4, showing a number of mechanical resonances in the frequency range from 1 MHz to 100 MHz. The strongest resonance, which from mechanical FEM simulations of the microtoroid can be identified as the fundamental flexural mode (FFM), was chosen for the feedback cooling experiments.

Supplementary Figure 5 shows a zoom in on the FFM and the Lorentzian fit yields a mechanical resonance frequency of $\Omega_\mathrm{m}/2\pi = 6.13\,\mathrm{MHz}$ and a linewidth of $\Gamma_\mathrm{m}/2\pi = 14\,\mathrm{kHz}$. The mechanical quality factor can then be found to be

$$Q_\mathrm{m} = \frac{\Omega_\mathrm{m}}{\Gamma_\mathrm{m}} = \frac{6.13\,\mathrm{MHz}}{14\,\mathrm{kHz}} = 437. \tag{27}$$

In order to calibrate the measured spectrum in terms of absolute mechanical displacement we utilize that in the bad cavity regime and in the limit of negligible radiation pressure backaction heating the absolute measurement noise level $S_{xx}$ is related to the optomechanical cooperativity $C$ as [13]:

$$S_{xx} = \frac{x_\mathrm{zpf}^2}{4\eta\Gamma_\mathrm{m}C}, \tag{28}$$

where $\eta = \eta_\mathrm{c}\cdot\eta_\mathrm{taper}\cdot\eta_\mathrm{mm}\cdot\eta_\mathrm{qe}$ is the total optical detection efficiency, $\eta_\mathrm{c}$ the coupling efficiency and $x_\mathrm{zpf} = \sqrt{\hbar/2m_\mathrm{eff}\Omega_\mathrm{m}}$ the mechanical zero-point fluctuation amplitude. In case of shot noise limited homodyne detection, the cooperativity is proportional to the SNR:

$$C = \frac{\mathrm{SNR}V_\mathrm{d}}{8\eta n_\mathrm{th}}, \tag{29}$$

where $V_\mathrm{d}$ is the detected variance and $n_\mathrm{th}$ is the thermal occupancy of the mechanical mode which, in the relevant high temperature limit, is given by $n_\mathrm{th} \approx k_\mathrm{B}T/\hbar\Omega_\mathrm{m}$.

Using the above quoted numerical values, the SNR from Supplementary Figure 4 of 22.4 dB, and an FEM simulated effective mass for the FFM mode of $m_\mathrm{eff} = 10\,\mu\mathrm{g}$, we find: $x_\mathrm{zpf} = 11.6\,\mathrm{am}$,

$n_{\text{th}} = 1.0 \cdot 10^6$, $C = 2.5 \cdot 10^{-5}$, and $\eta = 0.019$. Finally, the mechanical displacement sensitivity is found to be $\sqrt{S_{xx}} = 14.63\,\text{am}/\sqrt{\text{Hz}}$. The correspondingly calibrated transduction spectrum for the FFM mode is plotted in Supplementary Figure 5.

## D. Supplementary Method 4: Feedback circuit

The electric circuit used for feedback actuation of the mechanical resonator is illustrated in Supplementary Figure 6. The input AC-coupled homodyne photocurrent was first band-pass filtered by a combination of high-pass and low-pass filters and then split into feedback and monitor / acquisition signals. The feedback signal was subsequently amplified in two stages providing 10 and 30 dB gain respectively. A variable signal delay was used to introduce the crucial 90° delay of the feedback force with respect to the mechanical motion and two variable 10 dB attenuators were used to control the electrical feedback gain. Finally, a bias-tee, modified to accept up to 300 V DC input, was used to add a bias voltage to the feedback electrode.

### 1. *Circuit characterisation*

Ideally, a flat frequency response of the feedback circuit is desired in the frequency band covering the mechanical resonance. To confirm that this was the case the transfer function of the employed combination of filters and amplifiers was measured using an electronic network analyser (Agilent E5061B). Supplementary Figure 7 shows the measured complex voltage transfer function ($S_{21}$ parameter) of the feedback circuit.

## E. Supplementary Method 5: Feedback gain calibration and temperature inference

Theoretically, the effect of the implemented feedback cooling technique is varied by the feedback gain $G_{\text{Fb}}$, cf. Supplementary Note II A 2. However, experimentally, the controlled quantity is the electric gain given by the implementation of the feedback circuit. When the delay of the feedback signal is appropriately set for generating a dissipative cooling force, the feedback gain is related to the cooled mechanical resonator linewidth $\Gamma_{\text{Fb}}$ by $G_{\text{Fb}} = \Gamma_{\text{Fb}}/\Gamma_{\text{m}} - 1$, which allows to deduce $G_{\text{Fb}}$ from the transduced mechanical spectra as a function of the applied electric gain.

However, this approach breaks down for flat spectra, as $\Gamma_{\text{Fb}}$ takes on very large values. Instead, we fit the measured spectra to the model given by Supplementary Equation (9), where the gain $G_{\text{Fb}}$, the resonance frequency $\Omega_{\text{m}}$ and the measurement noise $S_{x_{\text{n}}}$ are taken as fit parameters.

Given these parameters, the integral of the spectrum defined by Supplementary Equation (8) can be used to determine the temperature [5]:

$$T_{\mathrm{Fb}} = \frac{m_{\mathrm{eff}}\Omega_{\mathrm{m}}^2}{k_{\mathrm{B}}2\pi} \int S_x(\Omega)\mathrm{d}\Omega, \tag{30}$$

where $k_{\mathrm{B}}$ denotes the Boltzmann constant.

In addition to extracting the temperature from the fits, we also deduced the feedback gain ($G_{\mathrm{Fb}}$) for different settings of the electronic gain. As it is illustrated in Supplementary Figure 8, the feedback loop exhibited a nonlinear behaviour. This behaviour was due to a slight saturation of the feedback amplifier caused by the amplification of adjacent mechanical modes. The amplification of adjacent modes was due to the varying phase response of the feedback loop (cf. Supplementary Figure 7) which lead to amplification of side modes while cooling the central mode. It is however important to stress that such a nonlinear behaviour of the feedback loop was not affecting our measurement and characterisation of the mechanical modes; it merely reduced the feedback gain. The characterisation of the cooled mechanical mode (shown e.g. in Supplementary Figure 5) was obtained using a linear detector which had been rigorously characterised. So there is a linear relationship between the optical amplitudes and the electronic currents used for characterisation. We stress that, as explained in the preceding paragraph, the information provided by Supplementary Figure 8 was not used as a calibration for estimating the temperature.

The different gain behaviours for the coherent state and squeezed states were caused by different excitation levels when these two measurements were carried out (and were therefore not associated with the different quantum features). However, we note once more that this was not used as a calibration for estimating the temperature.

In Supplementary Figure 9 we present the effect of squashing.

### 1. *Uncertainty propagation on the temperature estimate*

To infer the out-of-loop temperature shown in Figure 3 in the main text, we evaluated Supplementary Equation (30) as explained above. The SNR was determined from the linear spectral density (LSD) when no feedback was applied. More precise, the SNR was derived by a fit to the data, using

$$f(\nu) = a + b\nu + F\frac{(\Gamma/2)^2}{(\nu-\Omega)^2 + (\Gamma/2)^2}, \tag{31}$$

i.e. the Cauchy ("Lorentzian") probability density function with a linear term as an offset. The function is defined such that $\Gamma$ yields the full-width-half-maximum value.

In total, seven parameters $(a, b, F, \Gamma_\mathrm{m}, \Omega_\mathrm{m}, G_\mathrm{Fb}, S_{x_\mathrm{n}})$, extracted by a non-linear fit, determine the temperature. Furthermore, we consider an uncertainty of 10 % on the knowledge of the effective mass and an uncertainty of 0.1 K on the initial temperature. From the fit routine, uncertainties on the previously named parameters were estimated. Upon these results, the standard deviation of the temperature estimate has been calculated according to

$$\Delta T(\Gamma_\mathrm{m}, \Delta\Gamma_\mathrm{m}, \Omega_\mathrm{m}, \Delta\Omega_\mathrm{m}, \dots) = \sqrt{\frac{\partial T}{\partial \Gamma_\mathrm{m}}^2 \Delta\Gamma_\mathrm{m}^2 + \cdots}, \tag{32}$$

where a prepended '$\Delta$' denotes the uncertainty estimate of the respective quantity.

### F. Supplementary Method 6: Data acquisition

The data presented in the manuscript was recorded by sampling the AC-coupled and bandpass filtered homodyne photocurrent using a digital sampling oscilloscope (LeCroy HDO6034). The signal was sampled at a rate of 50 MS/s yielding a Nyquist frequency of 25 MHz well above all relevant frequencies. For each measurement run the total sampling time was 100 ms resulting in 5 000 000 points per data set. In a subsequent post processing step the data was binned and the corresponding power spectra were derived by a numerical Fourier transformation.

For the recording of Figure 2(b) and (c) in the main text, the data was mixed down digitally at the resonance frequency $\Omega_\mathrm{m}$. The latter was determined beforehand by a fit. To acquire both $\langle \hat{X} \rangle$ and $\langle \hat{Y} \rangle$, we multiplied the data by $\sin(\Omega_\mathrm{m} t)$ and $\cos(\Omega_\mathrm{m} t)$. Next, a first order low-pass filter with a corner frequency of $\Gamma_\mathrm{m}$ cut off adjacent mechanical modes and decreased the contribution from shot noise.

## IV. SUPPLEMENTARY REFERENCES


[1] Mancini, S., Vitali, D. & Tombesi, P. Optomechanical cooling of a macroscopic oscillator by homodyne feedback. *Physical Review Letters* **80**, 688–691 (1998).

[2] Cohadon, P. F., Heidmann, A. & Pinard, M. Cooling of a mirror by radiation pressure. *Physical Review Letters* **83**, 3174–3177 (1999).

[3] Kleckner, D. & Bouwmeester, D. Sub-kelvin optical cooling of a micromechanical resonator. *Nature* **444**, 75–78 (2006).

[4] Wilson, D. J. *et al.* Measurement-based control of a mechanical oscillator at its thermal decoherence rate. *Nature* **524**, 325–329 (2015).

[5] Poggio, M., Degen, C. L., Mamin, H. J. & Rugar, D. Feedback cooling of a cantilever's fundamental mode below 5 mk. *Physical Review Letters* **99**, 017201 (2007).

[6] Corbitt, T. *et al.* Optical dilution and feedback cooling of a gram-scale oscillator to 6.9 mk. *Physical Review Letters* **99**, 160801 (2007).

[7] LIGO Scientific Collaboration. Observation of a kilogram-scale oscillator near its quantum ground state. *New Journal of Physics* **11**, 073032 (2009).

[8] Li, T., Kheifets, S. & Raizen, M. G. Millikelvin cooling of an optically trapped microsphere in vacuum. *Nature Physics* **7**, 527–530 (2011).

[9] Lee, K. H., McRae, T. G., Harris, G. I., Knittel, J. & Bowen, W. P. Cooling and control of a cavity optoelectromechanical system. *Physical Review Letters* **104**, 123604 (2010).

[10] Purdy, T. P., Peterson, R. W. & Regal, C. A. Observation of radiation pressure shot noise on a macroscopic object. *Science* **339**, 801–804 (2013).

[11] Safavi-Naeini, A. H. *et al.* Squeezed light from a silicon micromechanical resonator. *Nature* **500**, 185–189 (2013).

[12] Peterson, R. W. *et al.* Laser cooling of a micromechanical membrane to the quantum backaction limit. *Physical Review Letters* **116**, 063601 (2016).

[13] Bowen, W. P. & Milburn, G. J. *Quantum Optomechanics* (CRC Press, 2016).
